# Annotations

**Published:** 1889-10-05

**Authors:** 


### Annotations.

A glance at the titles of some of the subjects to be discussed at the forthcoming meeting of the American Public Health Association, to be held at Brooklyn on the 22nd, 23rd, 24th, and 25th of the current month, will show how thoroughly American medical scientists are imbued and animated with the idea that preventive medicine has a great part to play in the future history of the civilised world. "Railway Sanitation," for example, is not familiar as a topic for discussion in this country; whilst in certain parts of the continent of Europe sanitation conceived in the interests of railway passengers would seem to be a thing altogether unknown. The odours even of the waiting-rooms on some continental lines are unspeakable, whilst the disgusting abominations of the retiring-rooms are beyond measure intolerable. But "Railway Sanitation" in its widest bearings is set down for discussion at the forthcoming meetings at Brooklyn, and it may be hoped that some echo of the far-off discussion will reach the European continent and bear much-needed fruit there. "Methods of scientific cooking" are also in favour, and the medical men, of both sexes, among our American cousins will have abundant opportunity of expressing the convictions that are in them. The painful question suggests itself, Have the American doctors really any convictions at all on the subject of scientific cooking that are worthy to enter the field against the "rule of thumb" methods of the ordinary *chef*? For people will not eat to please science and scientists, but to please fashion and their palates. If science cannot make herself as agreeable at the table as the average cook does, then science may as well keep silence about "methods of scientific cooking." "Steamship Sanitation" is another subject which is ripe for consideration. It is impossible to understand why a floating town, "with all the sea before it where to choose," cannot get rid of its insanitary matters promptly, easily, and completely. Yet the sanitary offences committed on board even the best ships are almost as innumerable as they are unpardonable. It is to the shame of every ship's engineer that we are compelled to avow that ship sanitation is yet in its infancy. In all, there are ten subjects of primary importance set down for discussion. It is much to be desired that the scientists who read papers will give a practical turn to the proceedings in every instance. In this country, congresses and associations, like the Public Health Association, are beginning to be suspected both of insipidity and of practical impotency. The whole proceedings begin and end with talk, it is said; and with us, at any rate, talk followed by no results soon ceases to interest anybody. The world is beginning to understand the importance of sanitary questions; and medical scientists only need to display sufficient energy of enthusiasm and practical ability to give the subject an onward impetus, such as it has not previously had. We desire for these meetings and discussions the utmost possible success.

**Progressive Sanitation in America.**

The Rev. Arthur Tooth seems to have been fairly successful in his new *role* on Saturday last. The six or seven boys experimented upon yielded to the hypnotic or mesmeric influences as promptly as past generations of boys have done. The audience, including several medical men, seem to have been quite convinced, as previous audiences have been, that there was "something in it"; then apparently they separated, as former audiences have also done; and there, so far as we can see, the mtter ended. That there are more things in heaven and earth than are dreamed of in the physiological laboratory, we are well aware. Nevertheless, we confess to a liking for explanations wherever explanations are possible. It is desirable to know, for example, what Mr. Tooth thinks of his own powers, and what explanation he could give of them, if he would. It would also be satisfactory to know what he thinks of his boy performers. It would be most satisfactory of all to be told how hypnotism may be practically and honestly used in the amelioration of distressing symptoms, and in the cure of any class of human ills. To call mesmerism "humbug," "quackery," and the like is stupid. There is something in it; but what is the something? Can we get at it? Can we utilise it in any way? Or is the hypnotic power an endowment possessed only by a few, and capable of influencing only a few; and must it always remain a mere curiosity? It would seem so. There appears to us to be nothing more marvellous about it than about the power of thought possessed by the brain or the wonderful influence which the fervid orator or the passionate tragedian can exercise over a sympathetic audience. It is only more rare; certainly not more inexplicable. When the last word has been said about it, we are still exactly where we were at the outset. We do not understand it any more than we understand electricity, or lunacy, or love. But if it can be used for any practical and honest purpose, let it be used by all means. We are all for dismissing and destroying prejudice of every kind, and letting everything new and old that is not injurious have the fairest possible field and no favour. Mr. Tooth has some kind of impressions on the subject. Can he not put them into words?

**Mr. Tooth as a Hypnotist.**

Very captious critics might say that such a thing as a "scientific delusion" is an impossibility. If a thing is a delusion it is not scientific. But the word "scientific" is used in a hundred senses, and may therefore be permitted to stand where it is as a qualifying adjective to "delusion." The particular delusion to which we desire to call attention is of an amiable and harmless kind, and is, moreover, of some antiquity. We had thought indeed that it had died a natural death; but to our extreme surprise it was resuscitated at the closing meeting of the British Association by no less a person than the president, Professor Flower, himself. The distinguished professor said it was particularly gratifying to find pure or abstract science recognised among practical people—which is all very nice; but he went on to add that "nearly all the marvellous benefits which have been conferred on man by the application of scientific discovery or scientific knowledge have been the result of the discoveries of philosophers who are pursuing knowledge solely for its own sake—without any hope of reward, without any hope of its benefiting, perhaps, themselves or others, and very often amid the indifference, the neglect, and even the scorn of their contemporaries." No doubt "philosophers" love to lay the flattering unction to their souls that was laid to his by a certain well-known person in the New Testament parable, who thanked Providence that he was "not as other men," and particularly "not as this publican." And equally, no doubt, there have been "philosophers" who have been so much in love with science that they have pursued it at a loss, and have been scorned for their pains. But that is surely not a very exceptional thing in the world. We need not go so far back as to the early Christian martyrs to find examples of that kind. Your Salvation Army drummer glories in doing his work solely for its own sake; and if it brings upon him the scorn and even the head-breaking practices of his contemporaries, so much the better does he like it. As a matter of fact, the average scientist takes up science as a calling to live by exactly as the baker takes up baking and the prizefighter pugilism; and he does it for one of two reasons, either that his opportunities lie that way, or that his likings are in that direction. Does anyone believe for a moment, for example, that either Aristotle or Lord Bacon in remoter times, or Thomas Henry Huxley or John Tyndall in our own, chose science purely for the love of it, and without any fame or bread and butter object at all, any more than he believes that any of those distinguished persons were or are withered up by the scorn and neglect of their contemporaries? If we mistake not, all those gentlemen have loved or do love to live in the minds of their contemporaries, and equally admire the idea of being remembered after death by posterity. We do not for a moment say that scientists are less disinterested than other persons; neither do we admit that they are more so. The real truth is that disinterestedness has nothing to do with a man's profession at all, but is peculiar to the man. It is possible that, other things being equal, there is as large a proportion of disinterested individuals in the class of costermongers as there is in the class of scientists or of bishops. It was sound advice that was addressed to the modern world when truth seekers were imperatively instructed to "clear their minds of cant." No one admires scientists more than the present writer, and it is his admiration for them that makes him wish to see them preserving the same plain and truthful simplicity on the platform that they practise in the laboratory.

**A Scientific Delusion.**

